# Neural Decoding for Intracortical Brain–Computer Interfaces

**DOI:** 10.34133/cbsystems.0044

**Published:** 2023-07-28

**Authors:** Yuanrui Dong, Shirong Wang, Qiang Huang, Rune W. Berg, Guanghui Li, Jiping He

**Affiliations:** ^1^ School of Mechatronical Engineering and Beijing Advanced Innovation Center for Intelligent Robots, Beijing Institute of Technology, Beijing 100081, China.; ^2^ Department of Neuroscience, University of Copenhagen, Copenhagen 2200, Denmark.

## Abstract

Brain–computer interfaces have revolutionized the field of neuroscience by providing a solution for paralyzed patients to control external devices and improve the quality of daily life. To accurately and stably control effectors, it is important for decoders to recognize an individual's motor intention from neural activity either by noninvasive or intracortical neural recording. Intracortical recording is an invasive way of measuring neural electrical activity with high temporal and spatial resolution. Herein, we review recent developments in neural signal decoding methods for intracortical brain–computer interfaces. These methods have achieved good performance in analyzing neural activity and controlling robots and prostheses in nonhuman primates and humans. For more complex paradigms in motor rehabilitation or other clinical applications, there remains more space for further improvements of decoders.

## Introduction

**Fig. 1.**
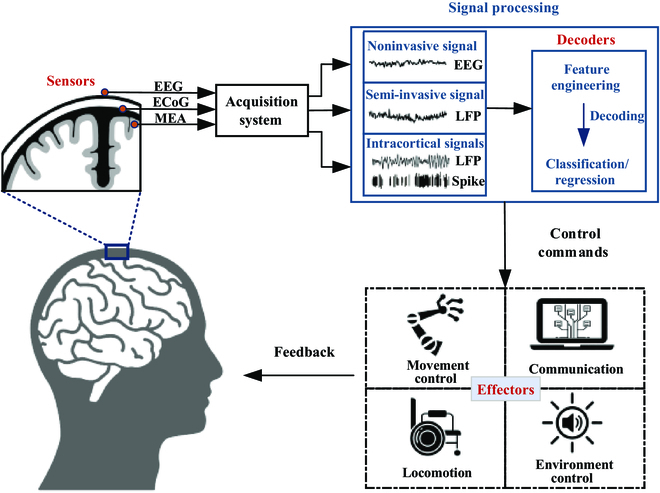
General paradigm of BCIs. Sensors collected neural activities, and signals are processed by the decoders to control external devices, which also return feedback to the individual.

Brain–computer interfaces (BCIs) provide a way for paralyzed patients to interact with external devices or restore sensory and motor function by translating neural activities obtained from the brain into control commands [1–5]. The BCI system consists of sensors, decoders, and effectors. Depending on the signal recording electrodes, there are mainly 3 types of BCIs: noninvasive, semi-invasive, and invasive BCIs. The paradigm of BCIs is shown in Fig. 1. In general, the sensors or electrodes record the neural activity, and the obtained brain signals are collected by the acquisition system, and then designed algorithms (decoders) are applied to extract features or patterns of specific signals, which will be translated into command instructions. Finally, the external device (the effectors) executes the task according to the instruction and feedback to the individuals.

Recently, intracortical brain–computer interfaces (iBCIs) have been applied in nonhuman primates (NHPs) and clinical rehabilitation applications [6–10]. The iBCIs collect neural electrical activity by surgically implanting a microelectrode array (MEA) into the related brain area. In motor-related iBCI, most electrodes are implanted in the motor cortex, and electrodes are also implanted in related brain regions, such as the somatosensory cortex (S1) and posterior parietal cortex [11,12], which provides more useful information for decoding movements. There are usually 2 types of iBCI—open-loop and closed-loop systems—depending on whether the external device provides feedback to the individual. For NHPs, open-loop iBCIs are the most commonly used paradigm. For example, MEAs were implanted into the cortex area related to movements of the rhesus monkey to control robots or prostheses, without the feedback from the external device to the brain during the execution of the task. On the contrary, the closed-loop iBCI is based on feedback from external devices, which is beneficial to real-time adjustments of the control system.

In the motor-related iBCI system, it is critical to correctly understand the motor intention and translate it to manipulate effectors. Typically, the neural signal decoding pipeline usually consists of feature engineering, decoder, and calibration [13], and the final output to the effector. Feature engineering aims to construct features, which represent task-related neural activities. The decoder is adopted to establish mapping relationships between the brain and behavior and translate it into the instruction for the effector. Due to the instability and variability of intracortical signals in the long-term recording, decoder calibration is necessary for the iBCI system. Thus, the decoder can be adjusted to make the system operate accurately and stably for paralyzed patients in clinical rehabilitation.

In this work, we summarized decoding methods of recent progress in iBCIs, focused on decoding upper limb movements. We constructed it into feature engineering, decoding approaches, and decoder calibration for motor-related iBCIs in detail. We discuss how these innovations improve the performance of iBCIs.

## Feature Engineering

The initial step in decoding is feature engineering, which involves extracting useful neural information from recorded intracortical signals. The objective of feature engineering is to enhance the accuracy of predicted models. Therefore, constructing the neural signal into representative features is crucial for developing a well-trained decoder. High-density MEAs, like Utah electrodes [14], are sensors that record extracellular neural signals implanted in NHPs and humans. Typically, an MEA can reach up to 100 channels, each of which records neural activities from 1 neuron or population of neurons [15]. Extracellular recordings encompass spikes and local field potentials (LFPs). The extraction of features from these 2 types of signals has been successfully applied to decoding intracortical signals in iBCI systems. The characteristics of representative features are depicted in Table 1.

**Table 1.** The characteristics of representative features.

Feature	Band	Characteristics	Advantages	Disadvantages	Application
Spike [16]	250 Hz–5 kHz	Frequency	Fast signal transmission speed, high accuracy	Easily affected by the number, location, and change of neurons, requiring high-frequency sampling	Reach-to-grasp task
NAV [57]	250 Hz–6 kHz	Temporal and spatial	High information, high accuracy, high reliability	High signal processing and computing cost requirements	Reaching and grasping tasks
SBP [25]	300 Hz–1 kHz	Highly spatial specificity	Very robust, high signal-to-noise ratio, low signal processing requirements	Relatively low information, cannot capture the temporal information of neural activity	Multiple finger kinematics
MWP [18]	Full band	Spectral and temporal	High information, high sensitivity, low requirement for the number and location of neurons	Affected by neuron types and densities, high signal processing requirements	Discrete imagined hand movements
LMP [29]	<300 Hz	Temporal	High signal stability, containing the overall characteristics of neuronal population activity	Relatively low spatial resolution and inability to record the activity of single neuron	Point-to-point task and instructed delay reach-to-grasp task

Spike is the electrical signal that best reflects the neural activity of the brain, and it has a high signal-to-noise ratio as well as high temporal and spatial resolution. Therefore, the firing pattern of spikes can be utilized to extract motor-related information effectively, which has been widely applied in iBCIs. Generally, the raw neural signal is bandwidth filtered (250 to 5,000 Hz), and spikes are detected using the threshold crossing method [16]. The spike firing rates are obtained by counting the spikes within a time bin of 30 to 100 ms, which are then used as inputs for the decoder. Furthermore, for each trial, the binned firing rate from all neurons can be concatenated together and organized into a neural activity vector (NAV) feature. It conveys spatial and temporal information of a neural representation, which is useful for decoding movements. Finally, signals from multiple trials are constructed as NAV features [17].

Mean wavelet power (MWP) is a neural feature obtained by calculating the mean of the standardized wavelet coefficients for each channel through wavelet decomposition, which can provide both frequency and temporal information of brain signals. MWP features of raw data can be further divided into 3 subfrequency bands—low-frequency MWP (0 to 234 Hz), mid-frequency MWP (mf-MWP, 234 Hz to 3.75 kHz), and high-frequency MWP (>3.75 kHz)—all of which stably tracked neural information after up to 3 years of recordings in a human with tetraplegia [18]. It has been demonstrated that mf-MWP contains enough information to be the optimal signal for predicting the imagined hand movements of the patient [19–24].

Spiking-band power (SBP), defined as the mean absolute value of neural activity at 300 to 1,000 Hz, is the low-energy band signal that has been found to correlate well with the firing rate of single or multiple cells with the largest amplitude on the electrodes. SBP maintains the spatial specificity of broadband spike-related features and has higher decoding performance than the threshold crossing rate [10,25]. It has been demonstrated that SBP is sufficient for predicting finger positions and velocities in closed-loop decoding.

In addition to spike-based iBCIs, recent studies have demonstrated the effectiveness of LFPs in decoding motor intention [26–28]. LFP is the signal obtained by the raw data with a low-frequency filter, with a band range of less than 300 Hz that hypothetically represents the population of neurons near the electrode. Local motor potential (LMP) features can be obtained by smoothing the time-domain amplitude of LFP, which has been shown to be the most predictive feature [29]. LMP feature can be directly fed into the end-to-end network for decoding hand kinematics [28]. Combining LMP with frequency-domain features, delta Hilbert envelope, achieves better performance than single LFP features in predicting hand kinematics [30].

It is important to note that different signal types may have different advantages and disadvantages in different application scenarios. Therefore, constructing a feature should be based on careful consideration of the specific application scenario. In the future, it is still to be further explored for the informative and low energy-consuming features.

## Approaches of Decoding Signals in iBCIs

In recent years, iBCI systems have generated many decoding algorithms for applications in both NHPs and paralyzed humans. The design of the decoder is crucial to meet the performance expectations of the end user, as it is closely related to the characteristics of input features and the tasks to be performed. Motor-related iBCI tasks can be divided into 2 categories: discrete and continuous movement. Decoders for discrete movements usually adopt classification algorithms to identify different tasks, while decoders for continuous movements need to predict the kinematics of the limbs by regression analysis of the signals. With the development of computer science, new techniques have found wider application in the decoding of signals for iBCIs. The exploration of new algorithms and the combination with new features are important for the development of iBCIs with low power consumption, high speed, and high performance. The algorithms for decoding discrete and continuous movement are described in detail below.

### Decoding for discrete movement

**Fig. 2.**
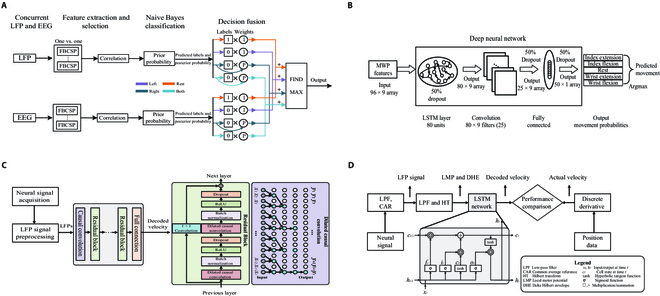
Architectures of neural decoding. (A) Naive Bayes models were trained separately on LFP and electroencephalogram (EEG) features, and the predicted kinematics were obtained through decision fusion and posterior probability from Bayes classifiers. (B) The DNN, consisting of LSTM and a convolutional layer, used MWP features in multiscale to predict movement. (C) TCN was used as the decoder for predicting kinematics. (D) The LFP-based decoder used LSTM, as illustrated in the gray-shaded block.

For discrete movements, the intracortical signals are decoded to recognize multicategory movements of arm extension grasp movements, upper limb wrist movements, and finger movements. The decoder learns the features of the training data to find the mapping relationship between the signal and the action category, and the classifier takes the corresponding category of the input signal. The most commonly used decoders in controlling robotic movement are linear discriminant analysis (LDA) [31], and naive Bayes decoder (Fig. 2A) [20,32]. Support vector machine (SVM) is a classical machine learning method that finds the maximum classification interval on the feature space and thus finds an optimal separation hyperplane to distinguish between different classes. For example, a male patient with spinal cord injury (SCI) performing a motor imagery task with 6 different hand, wrist, and finger motions, as well as in the presence of external stimuli to perform 6 or 7 different wrist and hand motions, combined WMP features with a nonlinear SVM classifier to analyze the signals and obtained good results for the recovery of voluntary movements in paralyzed patients [22,33,34].

With the development of machine learning, more complex networks have been developed in neural signal decoding for iBCIs. A convolutional neural network (CNN) is a multilayer neural network, which is widely used in computer vision. Recurrent neural network (RNN) and its variant long short-term memory (LSTM) are more suitable for processing natural language processing. For the neural signals collected from the tetraplegic participants performing 4-movement tasks, MWP features were extracted and a neural network deep neural network (DNN) was utilized to predict the discrete movements. DNN consists of LSTM and a convolutional layer, where LSTM is used to extract the temporal information from the signal, and the last layer of DNN uses an activation function for probabilistic prediction of the class, and the action with the highest probability value is the predicted movement at that time. DNN has a shorter response time as well as higher accuracy compared to other comparison algorithms, nonlinear LDA, SVM, and Bayes (Fig. 2B) [19]. The classification methods of decoding intracortical signals for discrete movements are shown in Table 2.

**Table 2.** The classification methods of decoding intracortical signals for discrete movements.

Ref.	Year	Subject	Feature	Decoder	Application
[31]	2022	A male with SCI	Spike	LDA	Cut and eat food in a complex bimanual self-feeding task
[34]	2019	Two rhesus macaques	Spike	SVM	One monkey for a motor task, 1 monkey for a sensory task
[22]	2018	A male with a C5-level SCI	MWP	SVM	Four-movement task
[24]	2020	A male with a C5-level SCI	MWP	SVM	Four-movement task
[57]	2018	Two rhesus macaques	NAV	Nonlinear SVM	Reaching and grasping tasks
[58]	2020	Two rhesus macaques	NAV	SVM	Reaching and grasping tasks
[32]	2022	A 72-year-old male	LFP, EEG	Naive Bayes	Four-class motor imagery tasks
[19]	2018	A male with C5-level AIS	MWP	DNN	Four-movement task

### Continuous kinematics decoding

IBCIs are the preferred systems for decoding continuous limb movements due to the rich motor-related information contained in the intracortical signals. Continuous kinematics control is generally realized by decoders to predict motion states, such as limb position and velocity. To meet this need, the Kalman filter uses the kinematic model as a basis to modify and optimize the output, and it has been widely used in offline, real-time, and clinical trials.

In continuous tasks, spike decoders have been widely used. Due to the long-time stability property of LFP compared to spikes, research on LFP-driven decoders has been increasing. However, early studies based on LFP usually used Kalman filters, which have lower performance than spike decoders [35]. The development of neural networks provides a more stable and robust approach for decoding continuous motor intention in both spike- and LFP- iBCIs. The temporal convolutional network (TCN) is a variant structure of the CNN network that uses 1-dimensional dilated causal convolutional layers and the features of LFP signals to achieve good performance in predicting hand movement (Fig. 2C) [28]. RNN is a method for processing sequential signals that can store the recent input representations into hidden states using feedback connections and thus has greater advantages in predicting continuous kinematics. However, traditional backpropagation for updating the hidden states can cause a gradient explosion or vanishing problem in RNNs. LSTM is a variant of RNN that can solve the gradient vanishing problem and improve the capture of long-term dependencies by introducing a gating structure [36]. Thus, LSTM has many applications in kinematic movement prediction [37,38]. Based on spikes, an LSTM model trained on multiday multielectrode recordings performed well for decoding intended cursor velocity from human motor cortex signals, and it substantially improved the bits-per-second metric in point-and-select cursor tasks compared to a Kalman filter [39]. The first combination of LFP signals and LSTM decoder achieved significantly better decoding performance than the Kalman filter based on LFP and spike in predicting hand kinematics prediction tasks, indicating that LFP-driven LSTM decoder can provide high decoding performance, robustness, and low power consumption for iBCI (Fig. 2D) [30].

Deep learning networks have a strong capability of feature extraction and can construct decoding models composed of multistructured networks that incorporate useful information. Since RNNs do not have the capability of parallel computing, quasi-recurrent neural network (QRNN) alternates convolutional layers and a minimalist recurrent pooling function, which combines the parallel computing capability of CNNs and the time-dependent capability of RNNs for learning sequential data [40]. QRNN was adopted for velocity decoding and outperformed other comparative algorithms such as standard RNN and LSTM [41]. Notably, velocity and position are encoded differently in the motor cortex, and thus, decoding motion and velocity separately has a positive impact on improving performance. Using a dual LSTM model with simultaneous decoding of motion and velocity, the speed–direction LSTM produced a more accurate assessment of upper limb kinematic variables compared to the velocity Kalman filter as well as the velocity LSTM [42]. Thus, applying RNN and its variants for intracortical signals can improve performance in decoding continuous movements. The approaches to decoding intracortical signals for continuous kinematic movements are summarized in Table 3.

**Table 3.** The approaches of decoding intracortical signals for continuous kinematic movements.

Ref	Year	Subject	Feature	Decoder	Comparison	Application
[28]	2019	A monkey	LFP	TCN	LSTM decoders driven by hand-crafted features	Continuous reaching tasks
[39]	2019	Participants in BrainGate	Spike	LSTM	KF	Point-and-select cursor tasks
[30]	2019	An adult male Rhesus macaque	LFP	LSTM	KF	Self-paced reaching tasks
[41]	2021	Three monkeys	Spike	QRNN	WF, WCF, Kalman filter KF, and UKF, SRNN, LSTM, GRU	One for a point-to-point task, 2 for delayed reach-to-grasp task
[42]	2019	A monkey	Spike	Speed-direction LSTM	Velocity KF and the velocity LSTM	Center-out task
[76]	2019	Four rhesus monkeys	Spike	LSTM	WF, KF, UKF	Center-out arm reaching, bimanual reaching, and bipedal walking on a treadmill
[43]	2021	Two macaca mulatta	Spike-LFP	MSF	LFP–PPF,spike-KF	Three-dimensional (3D) reach-and-grasp movement task

WF, Wiener filter; WCF, Wiener cascade filter; KF, Kalman filter; UKF, unscented Kalman filter; SRNN, standard recurrent neural network; GRU, gated recurrent unit; PPF, point process filter.

In current iBCI systems, the neural activity is often analyzed using a single signal scale, while in real systems, movements may be the outcome of the combined effect from multiple scales. To improve the representational relationship between neural activity and actual movement, it is crucial to construct decoders that extract task-relevant information at each scale. The recently developed multiscale filter model can decode spike-LFP signals across multiple scales, sessions, and monkeys by sharing their principal mode of low-dimensional dynamics. Compared to single-scale models, the multiscale dynamical model has shown better performance in decoding intracortical neural signals for continuous movements [43,44].

## Decoder Calibration

**Fig. 3.**
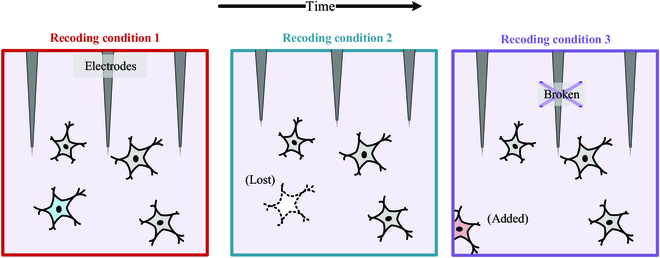
Neural variability due to electrode micromovement or broken. The neuron recorded by the electrode may be lost or added, which decreased the stability of the interface.

iBCIs can record intracortical neural signals for extended periods, which is beneficial for the mechanisms of motor control. Commonly, MEAs can work for about 1 to 3 years after implantation in NHPs [45,46] and have up to 5 years in humans [8,47]. During long-term recordings, the relationship between limb movements and neural signals used to predict limb movements varies dynamically over time. Although it has the potential to improve the relationship over time, decreased stability of the interface can be caused by decreased immunity of the tissue to the electrodes, changes in recorded neurons due to instability caused by micromovement, or breakage of the electrodes (Fig. 3) [48,49], so fixed decoders may not be suitable for iBCI systems over time. Therefore, maintaining the efficiency and stability of decoders is a critical issue in the development of long-term iBCI systems.

To address the issues, current researches mainly focus on 2 approaches to achieve rapid calibration of decoders. The first approach involves using automatic calibration methods to dynamically update decoder parameters without additional experimental steps. The other approach is to employ domain adaptation (DA) methods to reduce the reliance on current data and shorten the time needed to collect data.

### Automatic calibration method

To improve the stability performance of the decoder, the key point is to continuously learn the mapping between the neural signal and the predicted movement. Previous studies required participants to collect new data to calibrate the decoder before each experiment. Commonly, the training process requires multiple steps, and it needs to be gradually optimized to achieve good performance [1]. Additionally, the training of the subjects would be interrupted every 3 to 4 h of sessions, and data would need to be collected to calibrate the decoder before starting a new session [50]. A recalibrated feedback intention-trained (ReFIT) Kalman filter in closed-loop BCI is a 2-stage training approach, which has to be implemented at the beginning of each experiment day to control 1-dimensional index and medium-ring-little fingers by using spike or SBP features [25,51].

However, repeated calibration experiments would cost much time and increase the burden on patients, making the user experience less friendly and hindering the progress of rehabilitation training. Therefore, minimizing the calibration time or reducing the dependence on current data collection is essential for clinical application.

To save the extra data collection time brought by the calibration progress, an automatic decoder calibration can be constructed using naive Bayesian, or self-calibration, Kalman filter. In closed-loop BCI, the parameters of the Kalman filter with Bayesian regression methods were updated every 2 to 5 s, and decoder calibration was completed within 3 minutes, significantly reducing calibration time [7]. The Kalman decoder coefficients could be generated using an automatic calibration process, enabling on-demand access to high-performance iBCI technology at home [52]. A self-calibration Bayes classifier was proposed, which did not require daily calibration and the self-calibration classifier is accurate. Good performance was achieved on datasets spanning 48 and 58 d for offline analysis [53].

### DA

DA methods aim to reduce the disparities between 2 domains by learning knowledge from the source domain to the target domain [54]. In recent years, DA approaches have been widely used in computer vision and natural language processing. Specific to iBCI systems, the previous data could be regarded as the source domain, and the current data are the target domain. Currently, there are 2 main ways to implement data alignment in iBCI systems, one is to align the neural manifold space considering neural stationarity, and the other is to align the data distribution using data-driven DA approaches.

Recent studies have shown that neural function is built on specific population neural modes rather than on the independent modulation of individual neurons. Neural modes are termed neural manifolds, which are the main covariant patterns in neural populations [55]. It hypothesized that the latent dynamics underlying consistent behavior exist in low-dimensional neural manifolds that are relatively stable across days. Therefore, the alignment of low-dimensional neural manifolds can be used to stabilize neural activity. An approach based on canonical correlation analysis (CCA) is proposed to align data, compensate for recorded neuronal changes, and maintain decoder stability over long-term recordings [45]. To explore how the brain learns new skills and is influenced by experience, supervised recalibration utilized the same 2-stage decoder, consisting of a manifold-based stabilizer based on factor analysis and a BCI Kalman decoder for decoding continuous values of mouse velocity in real-time. The parameters of the stabilizer need to be updated in real-time considering the neural instability, while the decoder parameters are fixed. The method maintained BCI performance in 42 single-day and 2 multiday experiments (lasting 5 d each) [56]. The DA approaches for stabilizing the decoder are shown in Table 4.

**Table 4.** The domain adaptation approaches for stabilizing the decoder.

Ref.	Year	Subject	Feature	Alignment	Decoder	Application
[45]	2020	Six monkeys	Spike	CCA	Standard WF or naive Bayes	Two-dimensional center-out task
[56]	2021	Two rhesus macaques	Spike	Factor analysis	Velocity KF	Two-dimensional center-out cursor task
[57]	2018	Two rhesus macaques	NAV	PDA	Nonlinear SVM	Three-movement reaching and grasping tasks
[58]	2020	Two rhesus macaques	Spike	Symmetric uncertainty-based transfer learning	SVM	Three-movement reaching and grasping tasks
[57]	2020	Two rhesus macaques	Spike	PMDA	Nonlinear SVM	Three-movement reaching and grasping tasks
[19]	2018	Twenty-seven-year-old male with C5 AIS	mf-MWP	Transferred NN	DNN	Four-movement task
[60]	2019	One monkey	Spike	Adversarial domain adaptation network	LSTM layer + a linear layer	Center-out isometric wrist task
[61]	2023	One rhesus macaque	NAV	Adversarial discriminative domain adaptation	SVM	Three-movement reaching and grasping tasks
[62]	2022	Tetraplegia	Spike	DyEnsemble	Recursive Bayesian filtering	2D cursor
[38]	2021	Two monkeys	Spike	Constrained conditional LSTM GAN	LSTM	Reaching task
[63]	2023	One rhesus macaque	NAV	DDC	Two fully connected layers	Heterogeneous discrete reaching and grasping tasks

Data-driven DA approaches are another effective way to align data distribution for iBCI systems. From the perspective of data distribution, neural signals in long-term recording occur in distribution shifts over time. Therefore, aligning the current data with the historical data can reduce the distribution shift of the data and calibrate the decoder. Calibration with small current data could deal with the needs of large current data. By taking advantage of a large historical sample set, a principal component analysis-based domain adaptation (PDA) method was adopted to recalibrate the decoder with only ultra-small current samples, while reducing the calibration time [57]. Further, a symmetric uncertainty-based transfer learning method was proposed, which combined transfer learning with feature selection to reduce the demand for current data by selecting important and nonredundant features and the computational burden [58]. These above methods usually use a single source for decoder calibration. However, since the data corresponding to a single source may not be the best optimal for decoding the current data, this paper used the PCA-based multi-source domain adaptation method (PMDA), using multiple source domain data from multiple days to improve decoding accuracy. The PMDA algorithm can effectively utilize multi-source domain information by constructing a subclassifier and weight assignment scheme based on each source domain [59].

With the development of deep learning, new methods based on deep learning architecture for decoder calibration have been developed. The transferred neural network for the new task was obtained by the unsupervised updating method of DNNs using the MWP features of the intracortical signal. The decoder responds faster than SVM and sustains the performance without daily recalibration beyond a year [19]. An adversarial domain adaptation network was trained to match the empirical probability distribution of the reconstructed neural signal residuals. It decoded motor intention from low-dimensional latent representations of neural data and outperformed CCA and the minimization of a Kullback–Leibler divergence method with remarkably few data points [60]. The domain adaptation–decoder calibration framework adopted adversarial discriminative domain adaptation to learn the representation from source data so that the discriminator would not distinguish whether the data is from the source domain or the target domain. It extracted features of target data and put them into SVM, achieving well performance with a small amount of target data [61]. A dynamic integrated Bayesian filter (DyEnsemble) was proposed to handle neural diversity in online BMI control. DyEnsemble learned a pool of models containing multiple capabilities describing neural function and dynamic weights and assembled the models based on neural signals in a Bayesian framework. The method is a fully data-driven model with no strong assumptions about neural activity, and DyEnsemble coped with signal variability and improved the robustness of online control [62].

For the iBCI, most of the existing decoders can maintain good decoding results in the same task and the same subjects across sessions. However, in clinical applications, individual differences and different rehabilitation needs have made it essential for iBCI systems to derive more general algorithms for different experimental paradigms, different numbers of implanted electrodes, and cortical areas. Thus, it is challenging to maintain good performance of the decoder cross sessions, cross subjects, or cross tasks. In the latest study, using a generative model trained from a session on a monkey to synthesize new spike trains, a limited amount of extra real data can be rapidly adapted to new sessions or subjects. A generative model, the constrained conditional LSTM GAN, was used to learn the mapping between hand kinematics and neural spike trains based on the distribution characteristics of the data, thereby generating a large amount of new spike data. The decoder could be rapidly adapted to new sessions or subjects with a small amount of extra real data [38]. For the label shift problem caused by task changes, domain consensus clustering is utilized to map the clusters of unlabeled target data with the clusters of source domain data to solve the label shift problem caused by task changes. The decoder could achieve good performance under 2 heterogeneous scenarios, namely, partial domain adaption and open-set domain adaptation scenario [63].

## Applications in Restoration

The World Health Organization reported that up to 500,000 people worldwide suffer from SCI per year, which greatly affects the quality of life of paralyzed patients as the motor function of the limbs is limited. To explore the application of iBCIs in restoring motor functions, many studies have been conducted in NHPs and humans for upper limb movement [64]. The iBCI system enables the monkey to manipulate the robotic arm to perform the feeding task, representing a substantial development that connects animal brains directly with external devices. With further exploration of motor patterns, a high-speed BCI was first demonstrated in NHPs, capable of precisely controlling multiple fingers simultaneously, using real-time machine learning, which can separate the index finger from the middle, ring, or little finger to drive the prosthesis [10]. Generally, the iBCI systems have been applied in restoration, such as bidirectional iBCI, neuromodulated iBCI, and new experimental paradigms for more precise and multi-degree-of-freedom control of external devices.

### Bidirectional iBCI systems

To achieve more precise and naturalistic movements for rehabilitation movements, it may be necessary to feedback on the state of external devices to the brain. A bidirectional iBCI can provide sensory feedback information by electrically stimulating the sensory cortex while controlling the device through signals from the motor cortex of the brain [65]. By recognizing the motor intention of the intracortical signals in real time and thus mapping them to the parameter modulation of the stimulation system, the combined effect of the 2 makes the modulation of motor function more favorable to rehabilitation and has a more positive effect on clinical applications [8]. Therefore, bidirectional iBCI systems contribute to realizing closer to proprioceptive movement control.

Flesher et al. [66] performed experiments on a 28-year-old person with SCI by implanting 2 electrodes each in M1 and S1 areas, which could use intracortical microstimulation (ICMS) as a feedback source for closed-loop control of the prosthesis in a continuous 2-dimensional force matching task. The robotic prosthetic system was developed to establish a bidirectional BCI with sensory feedback that captures neural activity from the motor cortex of the brain to control the robotic arm, while sensors on the fingers of the robotic arm recode the mechanical forces they perceive and transmit them back to the somatosensory cortex via ICMS in the somatosensory cortex so that the user can feel the evoked sense of touch. The sensory encoding scheme of ICMS for the S1 electrode consisted of 2 components, variable intensity and multiple focal percepts, which evokes tactile sensations perceived as originating from locations on the hand. The success of somatosensory feedback is essential for skilled movement. In a further study, the patient was able to improve performance with the robotic limb by using the system. The time to perform the task on the upper limb was reduced by half, from a median time of 20.9 to 10.2 s. Evoked tactile signals effectively reduced the time to attempt to grasp objects, revealing that mimicking known biological control principles leads to task performance that more closely resembles able-bodied human abilities [8]. The iBCI system has been available for the patient for up to 7 years, and the electrodes are still working without other complications, providing hope for the clinical use of iBCI systems.

### Neuromodulated iBCIs

For patients with SCI, the pathway between the brain and muscles is broken, and functional electrical stimulation (FES) can be used to activate muscles to assist patients in performing movements. FES is an effective treatment to improve muscle activity by applying small currents of electrical stimulation to muscles or nerves, which can be used to control muscle contraction, thereby improving the patient's mobility. Both the intensity and duration of electrical stimulation have a direct effect on the muscle activity stimulated, and the combination with iBCI is important for the use of proprioceptive movements in SCI patients [67].

In a groundbreaking achievement, 2 intracortical MEAs were implanted in the hand region of the motor cortex of a 53-year-old male patient with SCI, and a total of 36 percutaneous electrodes were successively implanted in the right upper and lower arms to electrically stimulate muscles of his hand, elbow, and shoulder. This is the first time that iBCI has been combined with FES, where the patient uses his paralyzed arm and hand to coordinate reaching and grasping movements, activating the relevant muscles with the FES and controlling them with his intracortical signals through an iBCI [68].

Ganzer et al. integrated the iBCI and FES in the system for a 27-year-old patient with SCI to reconnect the brain to the paralyzed limb to restore function. Utah electrodes were implanted in M1 for recording intracortical neural signals, and the FES system consisted of a multichannel stimulator and a 130-channel cuff simulated electrode. This high-resolution cuff electrode was wrapped around the patient's forearm to control the patient's forearm movements by electrically stimulating the appropriate muscles, associating intracortical signals with muscle activation in real-time, and the upper limb could voluntarily perform 6 or 7 different wrist and hand motions [33]. Using the BCI-FES technology, the patient appropriately manipulated the Grasp and Release Test in real-time with a natural grip, advancing the decoding performance of the BCI-FES technology from a research device to a clinical neuroprosthesis [22]. In a recent study, residual subperceptual hand touch signals were demultiplexed from ongoing efferent motor intent in real time from signals collected in the M1 and used for both neurofeedback and prosthetic manipulation, resulting in closed-loop sensory feedback for intracortical control. This patient was able to control the already paralyzed upper limb for multiple levels of touch and grip strength tasks, achieving simultaneous recovery of motor function and haptics [24].

In addition, motor intention can be obtained by stimulating peripheral nerves, which are currently widely used in prosthetic control. Peripheral nerve signals consist of motor and sensory nerve fibers, and neural signals are characterized by high accuracy and large bandwidth. Two 100-channel Utah slanted electrode array (USEA) electrodes are implanted in the median and ulnar arm nerves of a human patient with upper limb amputation, and the participant could restore control of 5 degrees of freedom and sensation of up to 131 proprioceptive and cutaneous hand sensory percepts [69]. In other studies, participants reported tactile and cutaneous sensations when stimulating the sensory fascicle and deeper proprioception when stimulating the motor fascicle. The specific fascicles can be selectively simulated, and it is beneficial to create highly selective peripheral nerve interfaces [70]. Therefore, the combination of iBCI and FES on peripheral nerves is a feasible solution to restore motor and sensory function for the rehabilitation of SCI patients.

### Development of new paradigms

Bimanual arm movements are commonly required in daily life, yet most current studies have focused on the control of a single robotic arm. Thus, a bimanual BCI was developed to enable monkeys to directly control both robotic arms and perform continuous reaching movements with both hands [71]. In a recent study, 6 electrodes were implanted in the motor and sensory cortex of both sides of a paralyzed patient’s brain. After up to 9 months of training and learning, the paralyzed patient was able to complete self-feeding by controlling 2 robots at the same time. Future work will expand the application scenarios of iBCIs by developing bidirectional BCIs based on sensory feedback for bimanual arms control [31].

The ability of individual fingers to perform finer movements in grasping tasks and continuous motor control is an important feature of primates compared to the motor control of the entire upper limb. Finger movement control is more challenging and has greater significance for improving life quality. Therefore, the specific relationship between intracortical neural activity patterns and finger movements remains to be explored. An experimental paradigm for controlling finger movements through iBCIs was carried out on 2 rhesus macaques. They could effectively differentiate between index finger movements and middle-ring-little finger movements in online decoding with good results [25,51]. Recently, a real-time, high-speed BCI was developed that adopts real-time machine learning to predict finger velocities of 2 monkeys and can accurately control neural prosthesis by separating the index finger from the middle-ring-little finger group simultaneously [10]. For a paralyzed human, a linear mapping of torque sensor data from robotic finger motors to pulse sequence amplitudes of ICMS in S1 hand regions provided intuitive feedback about the intensity of force on each finger individually, with signals from the M1 area to close-loop control of the prosthesis [66]. Although most of the clinical applications of iBCI mostly are still in the early stage, the success of these studies demonstrates the potential applications of iBCI in restoration.

## Perspectives and Conclusions

In this review, we present the development and recent progress of decoding approaches in iBCI systems, which have promising potential for motor rehabilitation in patients with physical disabilities. However, neural decoding remains a significant challenge, which directly limits the application of iBCIs. Therefore, one of the key directions for iBCI is to simplify and accelerate the decoding methods. We propose the following plausible aspects to achieve this goal.

First, decoding algorithms are developed for high-density electrodes with hundreds of channels. Currently, implanted electrodes are evolving into multichannel, high-density, and high compatibilities, such as Neuropixel and its iterative versions, arrays of small and flexible electrode “threads”, up to thousands of channels [72–75]. However, the huge data stream and the bandwidth limitations make it difficult for decoders to process the collected data in real time while maintaining good performance. Therefore, fast and efficient online decoding of a large amount of data is a necessary and critical step for future clinical applications.

Second, the development of general decoders across sessions, tasks, and subjects. Since long-term recording, task changes, and subject changes make major challenges to the performance of the decoder, the construction of a stable and general decoder can be constructed from various aspects of feature engineering, decoding methods, and correction methods. Thus, exploring multiscale features and combining generative models with DA methods are feasible ways to build a more general iBCI system. It is important to acquire multiscale information and meet the demand for data to decode movements across sessions, across subjects, and across tasks.

Third, we need to develop decoding algorithms applicable to new experimental paradigms, such as bimanual robotic limbs, and the precise control of finger movements, which contribute to the mechanistic study of upper limb motor rehabilitation. Tactile feedback is introduced in neuromodulated iBCIs to achieve motor functions closer to proprioception, while expanding the applications of iBCIs also introduces more variables for the development of decoding algorithms and brings greater challenges to the performance of iBCIs. Therefore, we can exploit machine learning techniques to improve the real time and generalizability of the algorithms. New algorithms should consider the diversity of neural activities, as well as the complexity of the system, to improve the reliability and accuracy of iBCIs by better modeling the neural mechanisms of motor control. In addition, we can consider low energy features, optimization of decoding algorithms, and domain adaptation methods, which can greatly reduce the computational load and improve the decoding efficiency.

In conclusion, iBCIs have contributed significantly to the restoration of limb function for patients with physical disabilities, providing an alternative and promising way to improve their life quality. Recent studies in the laboratory and the clinic have shown that MEA-based BCIs control external devices, such as robotic arms, by recognizing the motor intention of people with disabilities. In the future, with the advances in multidisciplinary technologies such as engineering technology, material technology, machine learning, and interdisciplinary integration with neuroscience, there will be continuous development in sensors, decoding algorithms, and external device control. The neural decoding of the iBCIs together with all the above technologies will become more reliable in experiments and clinical tails and hopefully be applicable for motor rehabilitation at home.

## Data Availability

The original data supporting this review are from previously reported studies and datasets, which have been cited. The processed data are available from the corresponding author upon request.
